# Inhibition of NUCB2 suppresses the proliferation, migration, and invasion of rheumatoid arthritis synovial fibroblasts from patients with rheumatoid arthritis in vitro

**DOI:** 10.1186/s13018-022-03453-2

**Published:** 2022-12-30

**Authors:** Shuo Zhang, Tao Zhang, Yayun Xu, Genxiang Rong, Juehua Jing

**Affiliations:** 1 Department of Orthopedics, The Second Affiliated Hospital of Anhui Medical University, 678 Furong Road, Hefei, 230032 Anhui People’s Republic of China; 2 Inflammation and Immune Mediated Diseases Laboratory of Anhui Province, School of Pharmacy, Anhui Medical University, Hefei, China; 3 Department of Epidemiology and Biostatistics, School of Public Health, Anhui Medical University, Hefei, China; 4 Department of Orthopedics, The First Affiliated Hospital of Anhui Medical University, Hefei, China

**Keywords:** Rheumatoid arthritis, Synovial fibroblast, Nucleobindin-2, Synovium, Proliferation, Migration, Invasion

## Abstract

Rheumatoid arthritis (RA) is an autoimmune polyarthritis in which synovial fibroblasts (SF) play a major role in cartilage and bone destruction through tumorlike proliferation, migration, and invasion. Nesfatin-1, an 82-amino-acid-long peptide discovered by Oh-I in 2006, is derived from the precursor protein nucleobindin-2 (NUCB2). NUCB2/nesfatin-1 promotes cell proliferation, migration, and invasion in various tumors. We have previously shown that increased nesfatin-1 levels in the synovium may be associated with disease severity in patients with RA. However, the effect of NUCB2 on the tumorlike transformation of RASF has not yet been reported. The expression of *NUCB2* mRNA in the synovium of RA and non-RA patients was further confirmed using three individual datasets from the NCBI GEO database. Gene set enrichment analysis (GSEA) was employed to explore the association between *NUCB2* mRNA and RA-related gene signatures or signaling pathways in the GSE77298 dataset. Cell proliferation, migration, and invasion abilities were determined using Cell Counting Kit-8 (CCK-8), 5-ethynyl-2'-deoxyuridine (EdU), wound healing, and transwell assays, respectively. The results showed that the levels of *NUCB2* mRNA in the synovium were significantly elevated in patients with RA. Moreover, GSEA showed that high expression of *NUCB2* mRNA was related to gene signatures, including those involved in the cell cycle, DNA replication, extracellular matrix–receptor interaction, and focal adhesion. Furthermore, the results of CCK-8 and EdU assays indicated that inhibition of NUCB2 markedly repressed RASF proliferation. Additionally, the results of wound healing and transwell assays demonstrated that inhibition of NUCB2 significantly suppressed the migratory and invasive abilities of RASFs. Our findings are the first to demonstrate that the inhibition of NUCB2 suppresses the proliferation, migration, and invasion of RASFs in vitro.

## Introduction

Rheumatoid arthritis (RA), a common chronic autoimmune disease, is characterized by tumorlike abnormal excessive proliferation of joint synovial cells, angiogenesis, inflammatory cell infiltration, and bone destruction in various joints [1]. In patients with RA, the synovial lining thickness increases to 10–15 cell layers compared with the 1–3 cell layers observed in normal individuals [2, 3]. Synovial fibroblasts (SF) are unique cells that constitute the intimal lining of the synovium [4]. During the onset and development of RA, activated RASFs exhibit abnormal biological behavior and properties similar to those of cancerous cells, such as anchorage-independent growth, migration, and invasion, which promote RA progression and ultimately lead to joint destruction [5, 6]. Therefore, elucidating the mechanisms responsible for RASF proliferation, migration, and invasion may help identify potential therapeutic strategies for treating RA.

Nesfatin-1, an 82-amino-acid-long peptide discovered by Oh-I in 2006, is derived from the precursor protein nucleobindin-2 (NUCB2) [7]. The *NUCB2* gene encodes a 396 amino acid-long precursor peptide as well as a 24 amino acid-long signal peptide. It has been proposed that NUCB2 is cleaved into several fragments by prohormone/proprotein convertase (PC) 1/3 and PC2 to produce nesfatin-1 (amino acids 1–82), nesfatin-2 (amino acids 85–163), and nesfatin-3 (amino acids 166–396) [7]. It has been well demonstrated that nesfatin-1-mediated anorexigenic effects on the hypothalamus are key drivers of the metabolic action of NUCB2 [7]. To date, no significant biological activities have been detected for the C-terminal NUCB2-derived fragments, namely nesfatin-2 and nesfatin-3.

Numerous studies have indicated the critical role of NUCB2/nesfatin-1 in a variety of metabolic functions, including the regulation of food intake, glucose homeostasis, lipid metabolism, cardiovascular effects, and reproductive functions, as well as its possible involvement in psychological disorders [8, 9]. More recently, close links have been revealed between NUCB2/nesfatin-1 and tumorigenesis [10]. NUCB2/nesfatin-1 has been reported to promote cell proliferation, migration, and invasion in various cancers, including breast, colon, prostate, endometrial, thyroid, and bladder cancers [10]. Moreover, high expression of NUCB2/nesfatin-1 is associated with key traits of cancer as well as poor prognoses and outcomes [11]. Mechanistically, it has been shown that NUCB2/nesfatin-1 improved the migration, invasion, and EMT characteristics of colon and renal cancer cells through stimulation of the AMPK/TOC1/ZEB1 pathway [12, 13]. Takagi et al. found that after treatment of an endometrial cancer cell line with nesfatin-1, the mTOR protein was considerably phosphorylated, which improved cell proliferation and migratory capabilities [14]. However, a putative receptor for nesfatin-1 has not yet been identified. For better comprehension of the nesfatin-1 signaling pathways, identification, localization, and modulation of the nesfatin-1 receptor will be essential. We have previously shown that increased nesfatin-1 levels in the synovium may be associated with the severity of disease in patients with RA [15]. To the best of our knowledge, the effect of NUCB2 inhibition on the tumorlike transformation of RASF has not been reported.

Considering the tumorlike transformation of RASF during the pathogenesis of RA, together with the potential ability of nesfatin-1 to promote cell proliferation, migration, and invasion, we hypothesized that inhibition of NUCB2 may suppress the proliferation, migration, and invasion of RASFs. To test this hypothesis, the expression of *NUCB2* mRNA in the synovium in patients with RA and individual without non-RA was assessed. The association between *NUCB2* mRNA and RA-related gene signatures or signaling pathways was explored using gene set enrichment analysis (GSEA). The effect of NUCB2 inhibition on the proliferation, migration, and invasion of RASFs in vitro was investigated further.

## Materials and methods

### Patients and samples

Synovial samples were obtained from 3 RA patients who underwent joint replacement or synovectomy at the First Affiliated Hospital of Anhui Medical University. All enrolled patients with RA were required to satisfy the American College of Rheumatology (ACR)/European League Against Rheumatism 2010 classification criteria or the 1987 ACR criteria. The clinical details of the RA patients are provided in Table 1. All participants understood the purpose of this study and provided written informed consent prior to enrollment in this study, in accordance with the principles expressed in the Declaration of Helsinki. This study was approved by the Ethics Committee of Anhui Medical University.

**Table 1:** Demographic, clinical, and serological characteristics of specimens from RA patients

	RA patients
Male/female	0/3
Age (years)	57 [53, 64]
BMI (kg/m^2^)	19.4 [17.3, 21.5]
CRP (mg/l)	31.3 [7.5, 75.1]
ESR (mm/h)	53.3 [41, 65]
RF (U/ml)	750.8 [492.4, 1164.6]
Anti-CCP (RU/ml)	1146.3 [1085, 1200]
Duration of disease (years)	10.6 [10, 12]

*RA* rheumatoid arthritis, *BMI* Body Mass Index, *CRP* C-reactive protein, *ESR* erythrocyte sedimentation rate, *RF* rheumatoid factor, *anti-CCP* anti-cyclic citrullinated peptide. Values for non-normally distributed measurements were expressed as median (minimum, maximum)

### Source of the microarray data

Three gene expression profile datasets, namely GSE77298 [16], GSE55235 [17], and GSE1919 [18], were downloaded from the Gene Expression Omnibus (GEO) database (https://www.ncbi.nlm.nih.gov/geo). The GSE77298 dataset included 7 healthy control synovium samples and 16 RA synovium samples, the GSE55235 dataset included 10 healthy synovium samples and 10 RA synovium samples, and the GSE1919 dataset included 5 healthy synovium samples and 5 RA synovium samples. Platform and series matrix file(s) were downloaded from the GEO and saved as TXT files. The probes were transformed into the corresponding gene symbols according to the relevant annotation information on the platform. For gene symbols with multiple probes, the average value was taken as the final expression value. The average expression of *NUCB2* mRNA in synovial membrane samples from patients with RA and normal controls was compared.

### Gene set enrichment analysis (GSEA)

GSEA software v2.0.14x (JAVA version) was downloaded from the Broad Institute Gene Set Enrichment Analysis Web site (www.broad.mit.edu/gsea). The standard protocol was based on a previously published protocol [19]. Signature genes were extracted from Kyoto Encyclopedia of Genes and Genomes (KEGG) pathway enrichment. A false discovery rate < 0.05 and an adjusted *P* value < 0.05 were set as the cutoff criteria.

### Isolation and culture of synovial fibroblasts (SFs)

The isolation and culture of SFs were performed as described previously [20, 21]. Briefly, fresh synovium was cut into small pieces of approximately 1 mm^3^ in a sterile environment. The small pieces were then transferred to a cell culture flask containing Dulbecco's modified Eagle medium (DMEM) and 20% fetal bovine serum and cultured in an incubator. The cell culture flask was placed upright, incubated for 6 h at 37 °C in a 5% CO_2_ atmosphere, and then laid flat until the cells formed colonies. Subsequent cell experiments were conducted on cells between the third and sixth generations. The cell experiments were repeated three times. Three cell experiments were performed using RASFs isolated from three different RA patients.

### Lentivirus packaging and cell transfection

Adenoviral constructs carrying shRNA against *NUCB2* mRNA and control shRNA (negative control) were constructed by GenePharma Co. (Shanghai, China). The shRNA sequences against *NUCB2* mRNA used in this study were as follows: shRNA-NUCB2#1, AAGCTGTGCCTATTGACATAGAC; shRNA-NUCB2#2, AAGCAAAGAACTGGATTTAGTAA. For transfection, shRNA-NUCB2 NC, shRNA-NUCB2#1, and shRNA-NUCB2#2 were transfected into RASFs using Lipofectamine 3000 as the transfection reagent (Life Technologies, Inc., Carlsbad, CA, USA). At 48 h post-transfection, the cells were harvested, and quantitative real-time reverse transcription PCR (RT-qPCR) was conducted to determine the transfection efficiency.

### RT-qPCR assay

Total RNA was isolated from RASFs using TRIzol Reagent (Invitrogen, Carlsbad, CA, USA) according to the manufacturer's instructions and then reverse-transcribed into cDNA using a First Strand cDNA Synthesis Kit (Thermo Fisher Scientific, Waltham, MA, USA). The synthesized cDNA was used for RT-qPCR, which was performed using a CFX96 Real-time RT‒PCR detection system (Bio-Rad, USA) and a SYBR Premix Ex Taq Kit (TaKaRa Biotechnology, Tokyo, Japan) in a 25 µl volume for 40 cycles (15 s at 95 °C, 60 s at 62 °C, and 72 °C for 30 s). The Ct values of the samples were calculated, and the transcript levels were analyzed using the 2^−ΔΔCt^ method. The sequences of the primers were as follows: NUCB2: (forward) 5′-AAAGAAGAGCTACAACGTCA-3′ and (reverse) 5′-GTGGCTCAAACTTCAATTC-3′; GAPDH: (forward) 5'-GGAAAGCTGTGGCGTGAT-3' and (reverse) 5'-AAGGTGGAAGAATGGGAGTT-3'.

### Western blotting analysis

Cells were rinsed with ice-cold phosphate-buffered saline (PBS) and lysed using radioimmunoprecipitation assay (RIPA) buffer containing protease inhibitors and phosphatase inhibitors. A BCA Protein Assay Kit (Beyotime Biotechnology, Shanghai, China) was used to measure the total protein concentrations. Equal amounts of protein samples were prepared in loading buffer and boiled at 100 °C for 10 min, and then the boiled samples were electrophoretically separated by sodium dodecyl sulfate‒polyacrylamide gel electrophoresis (SDS‒PAGE) on a 10% gel and transferred onto polyvinylidene difluoride (PVDF) membranes (Millipore Corp, Billerica, MA, USA). After blocking with 5% nonfat dried milk in Tris-buffered saline (TBS) containing 1% Tween-20 for 2 h at room temperature, the membranes were incubated overnight with specific primary antibodies against NUCB2/nesfatin-1 (R&D Systems, # MAB5949) and β-actin (Abcam, #ab8227) at 4 °C overnight. After being washed three times with TBST, the membranes were incubated for 1 h at room temperature with horseradish peroxidase (HRP)-conjugated secondary antibodies (1:10,000 dilution). The protein bands were then detected in an enhanced chemiluminescence (ECL) detection system (Thermo Fisher Scientific Inc., Waltham, MA, USA), and quantification was performed using ImageJ software.

### Cell Counting Kit-8 (CCK-8) assay

A CCK-8 assay (Dojindo, Osaka, Japan) was used to assess the proliferative capacity of RASFs. RASFs were seeded in 96-well plates at a density of 5 × 10^3^ cells/well. Each well was treated with 10 μl of CCK-8 reagent and then incubated at 37 °C for 1 h. The absorbance of each well at 450 nm was then measured using a microplate reader (Bio-Rad, CA, USA). Cell proliferation was evaluated at different time points (6, 24, 48, 72, and 96 h).

### 5-Ethynyl-2ʹ-deoxyuridine (EdU) assay

To evaluate the proliferation viability of RASFs, an EdU assay was conducted using BeyoClick™ EdU-555 detection kits (Beyotime, Shanghai, China). Transfected RASFs were seeded in six-well plates and incubated in complete medium for 24 h. After incubation with 50 mM EdU for 6 h, the cells were fixed and stained for 30 min. Nucleic acid was stained using Hoechst 33342. All images were captured using a fluorescence microscope.

### Wound healing assay

For the wound healing assay, RASFs were seeded in six-well plates. When the cell confluency reached 90–100%, a sterile 200-μl pipette tip was used to make a straight scratch line on the monolayer of the confluent cells at the bottom of the culture plate; the cells were then cultured in basal medium without serum. Cell migration images were captured at time points of 0 and 24 h using an inverted microscope (Olympus Optical Co., Ltd., Tokyo, Japan). The total wound area was analyzed using ImageJ software to assess cell migration capacity.

### Transwell assay

For the transwell assay, RASFs were seeded into the upper chamber that was pretreated with or without Matrigel (Matrigel BD Biosciences, NY, USA); each chamber was loaded with 200 μl of serum-free culture medium and placed in 24-well tissue culture dishes. The lower chambers were filled with 800 μl of DMEM containing 20% FBS. After 24 h of incubation, the cells from the upper chamber were removed, and the invading cells were fixed and stained. All images were captured using a microscope (Olympus, Tokyo, Japan).

### Statistical analysis

The data were analyzed using SPSS (version 17.0; IBM Corp., Armonk, NY, USA). The data are expressed as the mean ± standard error of the mean (SEM), and statistical significance was set at *P* < 0.05. The normality of variables was evaluated using the Kolmogorov‒Smirnov normality test. The cell viability data from the CCK-8 assay were analyzed using repeated measures analysis of variance (ANOVA) followed by the least significant difference (LSD) test. Student’s *t* test for independent samples was used for comparisons between two groups. One-way ANOVA, followed by LSD post hoc test, was performed for three or more groups.

## Results

### *NUCB2* mRNA expression was significantly upregulated in the synovium of patients with RA compared with those of normal controls from GEO datasets

**Fig. 1:**
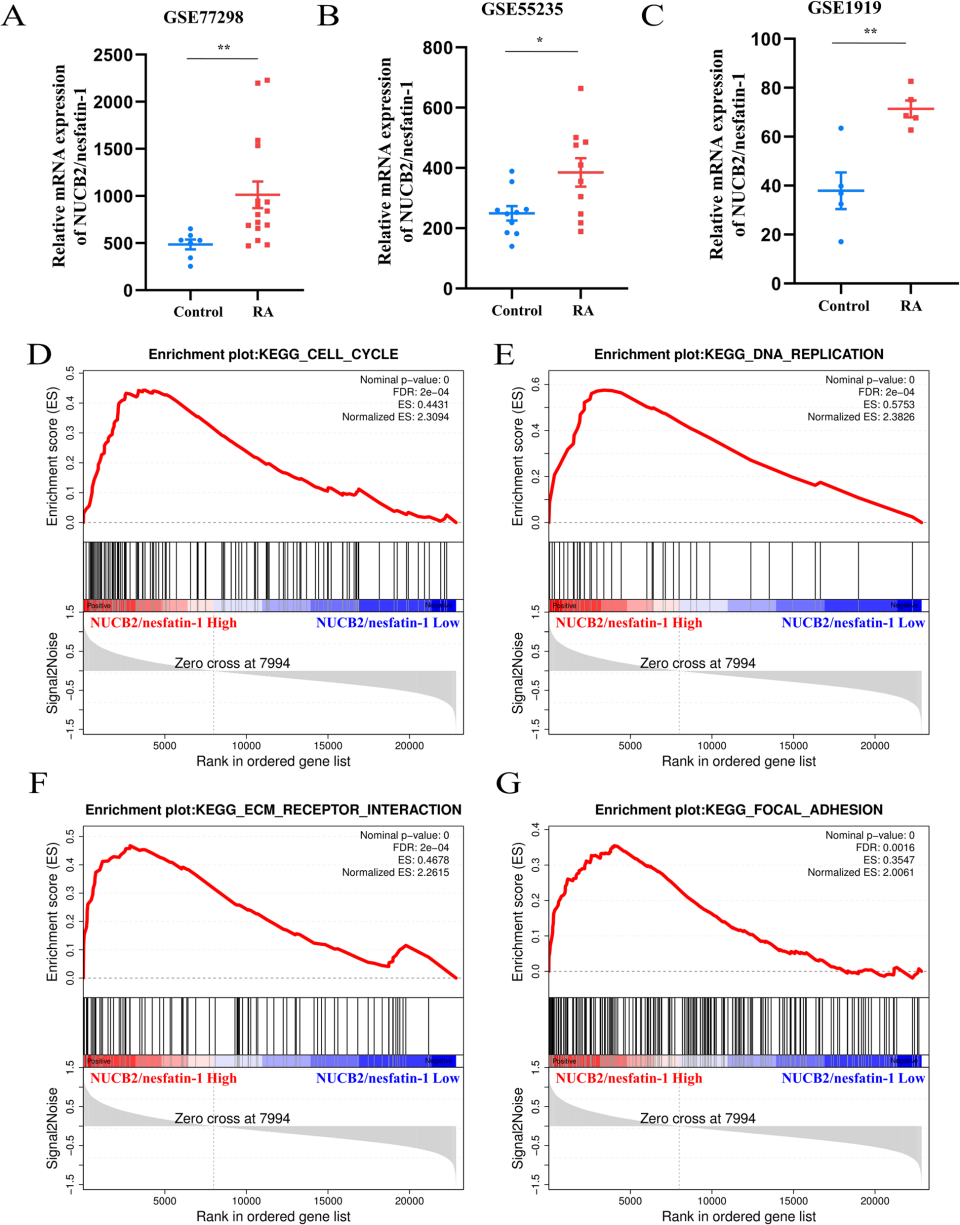
*NUCB2* mRNA expression in the synovium of patients with RA. **A**–**C** The expression level of *NUCB2* mRNA in the synovium of patients with RA was higher than that in normal controls (GSE77298, GSE55235, and GSE1919); **D**–**G** High expression of *NUCB2* mRNA was associated with the cell cycle, DNA replication, extracellular matrix (ECM)–receptor interaction, and focal adhesion in the GSE77298 dataset. All data are presented as the means ± SEMs. *P < 0.05, **P < 0.01 vs. control group

In the present study, the expression of *NUCB2* mRNA in the synovium of patients with RA was bioinformatically analyzed using three individual datasets from the NCBI GEO database (GSE77298, GSE55235, and GSE1919). Consistently, the expression of *NUCB2* mRNA in the synovium of patients with RA in GSE77298 (*P* < 0.01; Fig. 1A), GSE55235 (*P* < 0.05; Fig. 1B), and GSE1919 (*P* < 0.01; Fig. 1C) was higher than that in the synovium of normal controls according to the NCBI GEO database.

### High expression of *NUCB2* mRNA was associated with the cell cycle, DNA replication, ECM–receptor interaction, and focal adhesion in the synovium of patients with RA in the GSE77298 dataset

Next, gene set enrichment analysis (GSEA) was employed to explore the association between *NUCB2* mRNA and RA-related gene signatures or signaling pathways in the GSE77298 dataset. The results suggested that high expression of *NUCB2* mRNA was associated with gene signatures including genes involved in the cell cycle, DNA replication, extracellular matrix (ECM)–receptor interaction, and focal adhesion (Fig. 1D–G). These results suggested that NUCB2 may be involved in the regulation of RASF proliferation, migration, and invasion.

### Inhibition of NUCB2 repressed RASF proliferation

**Fig. 2:**
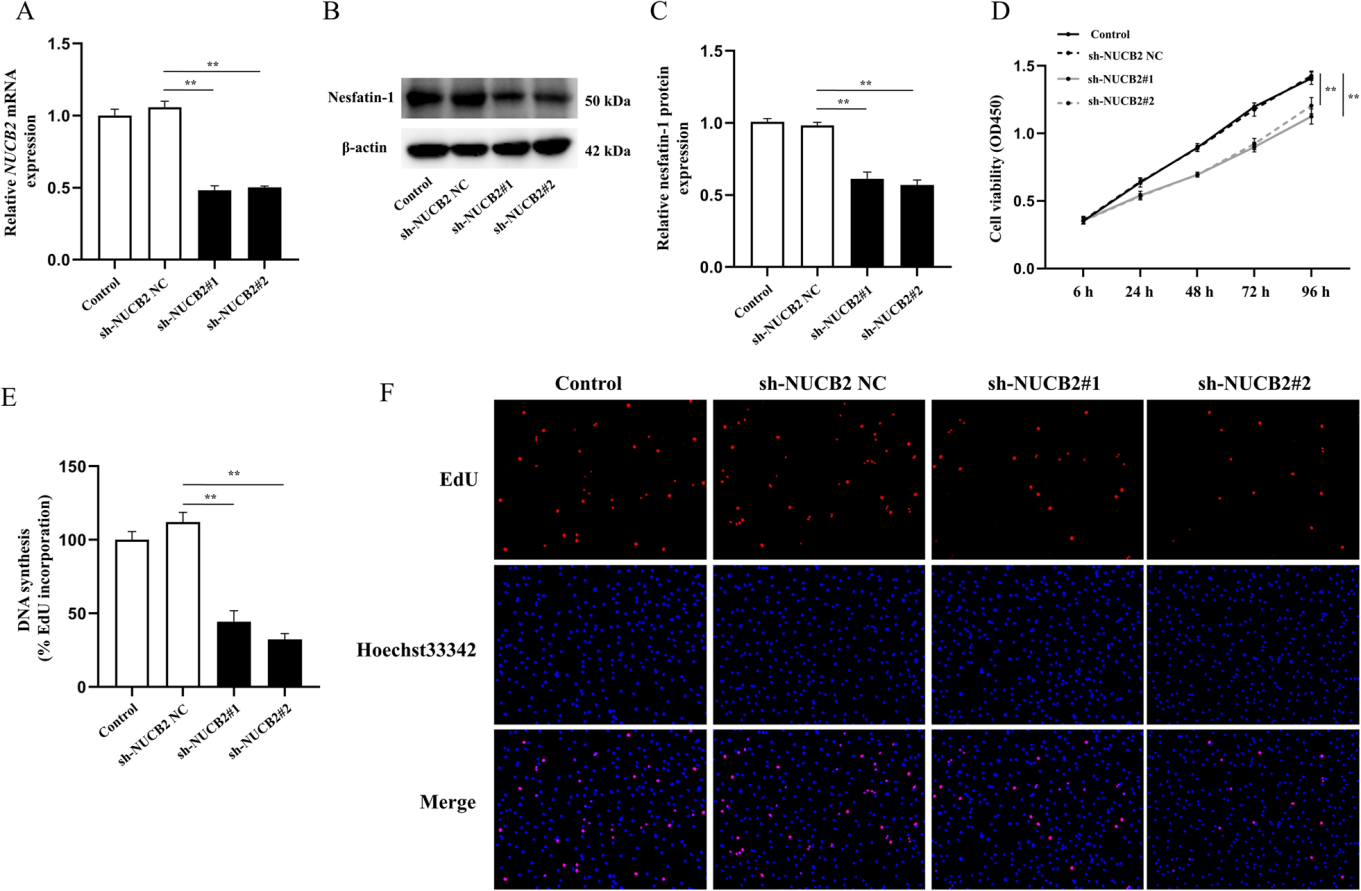
Effect of NUCB2 silencing on RASF proliferation. **A** RASFs were stably transfected with sh-NUCB2 NC, sh-NUCB2#1, and sh-NUCB2#2, and the mRNA level of *NUCB2* was analyzed using RT-qPCR. **B**, **C** The protein level of nesfatin-1 was analyzed using Western blotting. **D** The growth curves of cells were evaluated by CCK-8 assay after knocking down NUCB2 in RASFs. **E**, **F** An EdU assay was performed to evaluate cell proliferation. The samples were imaged at × 200 magnification. Three experiments were performed using RASFs isolated from three different RA patients, and a representative result is shown. **P < 0.01 vs. sh-NUCB2 NC group

To investigate the function of NUCB2 in RASFs, RASFs were stably transfected with sh-NUCB2 NC, sh-NUCB2#1, and sh-NUCB2#2. The ectopic expression of *NUCB2* mRNA and nesfatin-1 protein was confirmed using RT-qPCR (Fig. 2A) and Western blotting (Fig. 2B, C), respectively. The results showed that compared to sh-NUCB2 NC, sh-NUCB2#1 and sh-NUCB2#2 significantly decreased the mRNA expression of *NUCB2* (*P* < 0.01; Fig. 2A) and the protein expression of nesfatin-1 (*P* < 0.01; Fig. 2C). Inhibition of NUCB2 resulted in an obvious decrease in cell proliferation ability as determined by the CCK-8 assay (*P* < 0.01; Fig. 2D). Consistently, the results of the EdU assay also showed that NUCB2 silencing notably inhibited RASF proliferation (*P* < 0.01; Fig. 2E, F).

### Inhibition of NUCB2 suppressed the migration and invasion of RASFs

**Fig. 3:**
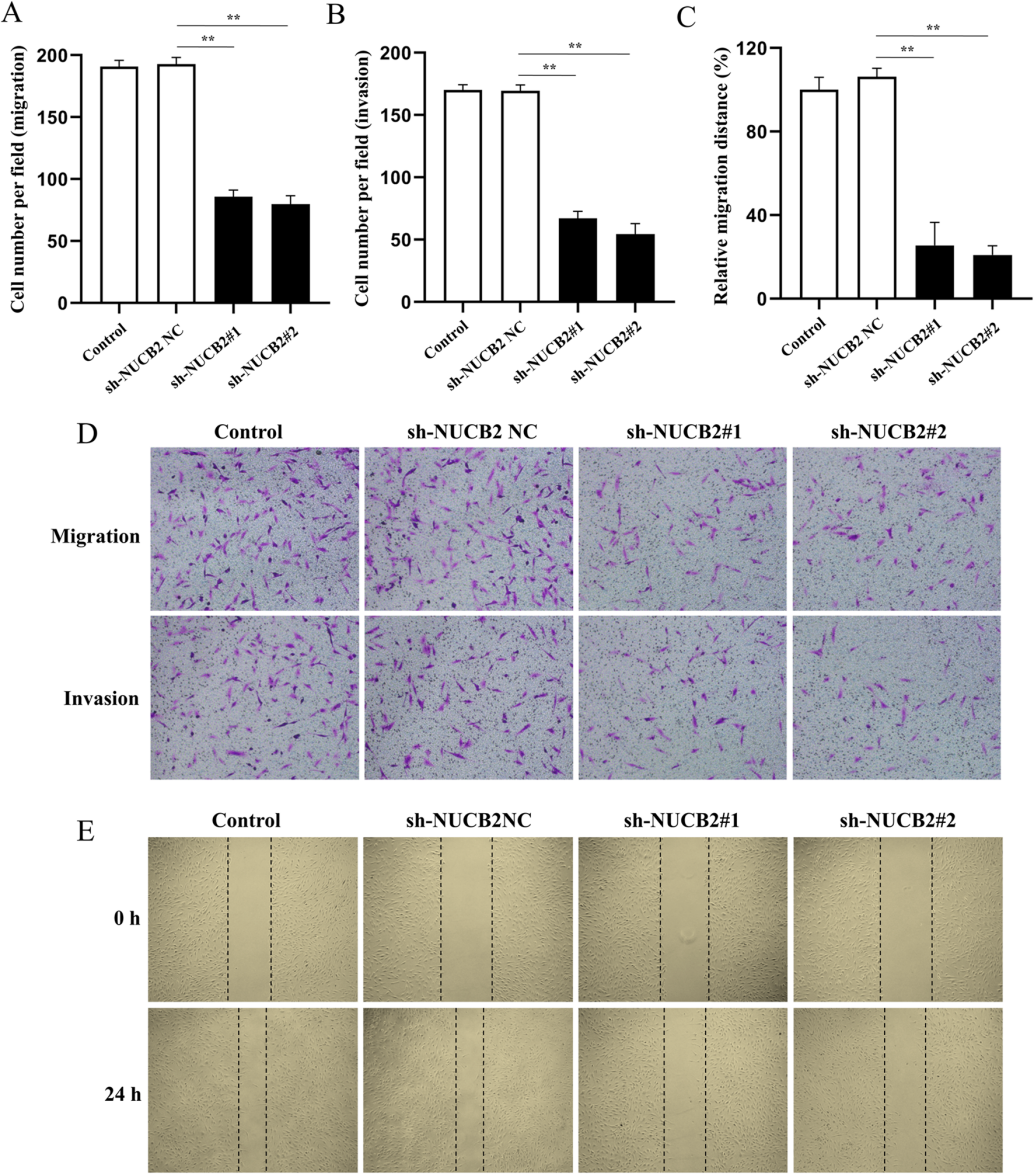
Effect of NUCB2 silencing on the migration and invasion of RASFs. **A**, **B**, **D** Transwell assays were performed to assess the migration and invasion of RASFs. The samples were imaged at × 100 magnification. **C**, **E** Cell migration was assessed using a wound healing assay. The samples were imaged at × 100 magnification. Three experiments were performed using RASFs isolated from three different RA patients, and a representative result is shown. **P < 0.01 vs. sh-NUCB2 NC group

As shown in Fig. 3A, B, D, NUCB2 knockdown in RASFs significantly prevented cell migration and invasion (*P* < 0.01), as assessed through transwell migration (without Matrigel) and transwell invasion (with Matrigel) assays, respectively. Similarly, the wound healing assay revealed a decreased migration rate after NUCB2 knockdown (*P* < 0.01; Fig. 3C, E).

## Discussion

To our knowledge, this is the first study to analyze the effect of NUCB2 on the tumorlike transformation of RASFs. Three main findings emerged from the present study. First, the levels of *NUCB2* mRNA in the synovium were significantly elevated in patients with RA. Second, high expression of *NUCB2* mRNA in the synovium of RA patients was related to gene signatures including genes involved in the cell cycle, DNA replication, ECM–receptor interaction, and focal adhesion, according to GSEA. Third, inhibition of NUCB2 markedly repressed the proliferation, migration, and invasion of RASFs.

There is growing evidence that adipocytokines have regulatory roles in the development of RA [22, 23]. It has been reported that the blood and synovial fluid (SF) levels of adiponectin, leptin, and resistin in RA patients are higher than those in controls [24–26]. Nesfatin-1, a novel adipocytokine derived from the precursor protein NUCB2, is widely expressed in both peripheral and central tissues [27]. A recent study showed a strong correlation between serum and SF nesfatin-1 concentrations and the severity of osteoarthritis, suggesting that nesfatin-1 may play a role in the pathophysiological changes that occur in osteoarthritis. In terms of RA, a higher serum nesfatin-1 level has been reported to be a characteristic of patients with a more severe clinical manifestation of RA [28]. Moreover, serum nesfatin-1 levels are associated with reduced atherosclerosis and increased plaque stability mediator levels in RA patients [29]. Our previous study indicated a positive relationship between the synovial nesfatin-1 concentration and serum RF level in patients with RA [15]. Notably, we were the first to report elevated NUCB2/nesfatin-1 levels in the synovium of patients with RA [12]. Consistent with the elevated NUCB2/nesfatin-1 levels in the synovium of patients with RA, we found that *NUCB2* mRNA expression was significantly increased in the synovium of patients with RA according to three datasets in the NCBI GEO database. These findings further confirm the abnormal expression of NUCB2/nesfatin-1 in the synovium of patients with RA and provide a basis for studying the role of nesfatin-1 in the synovium.

In our previous study, immunohistochemistry was used to assess the protein expression of NUCB2/nesfatin-1 in the synovium of patients with RA [15]. The results showed that NUCB2/nesfatin-1 was mainly expressed in the lining layers of the RA synovium, where synovial fibroblasts are located. The inflammatory cells (mostly lymphocytes and plasma cells) in the sublining layers showed little or no expression of NUCB2/nesfatin-1. Hence, the effect of NUCB2 on the tumorlike transformation of RASFs was investigated in the present study. Nevertheless, further studies are needed to assess the expression of NUCB2 in various types of cells in synovial tissue.

In recent years, the potential link between NUCB2/nesfatin-1 and tumorigenesis has been progressively revealed [11]. NUCB2/nesfatin-1 contributes to the development and progression of various cancers, such as prostate, colon, and breast cancers [30–32], and the underlying mechanism may be related to its ability to promote cell proliferation, migration, and invasion [33]. Specifically, it has been demonstrated that NUCB2 contributes to cancer metastasis and a number of related processes, including EMT [12, 13], endoplasmic reticulum (ER) stress [34], and cancer anorexia-cachexia syndrome [35], through regulation of the LKB1/AMPK/TORC1/ZEB1 and Akt/mTOR pathways. Given that RASFs exhibit tumorlike proliferation, migration, and invasion during the onset and development of RA [36], NUCB2/nesfatin-1 might exert regulatory effects on the tumorlike characteristics of RASFs. In the present study, analyses of the NUCB2-regulated gene signatures via GSEA using the GSE77298 dataset indicated that high expression of *NUCB2* mRNA was associated with the cell cycle, DNA replication, ECM–receptor interaction, and focal adhesion. The cell cycle and DNA replication are markers of cell proliferation [37]. It is well known that the ECM acts as a barrier to prevent tumor cells from invading and metastasizing [38, 39]. The formation of focal adhesions is essential for cell migration and invasion [40]. Therefore, the results of GSEA suggest that high expression of *NUCB2* mRNA may be associated with cell proliferation, migration, and invasion. The functional role of NUCB2 in the proliferation, migration, and invasion of RASFs was further evaluated by CCK-8, EdU, wound healing, and transwell assays. The specific shRNA targeting NUCB2 was designed based on the results of a previous study [41]. In that study, the protein expression of NUCB2 was dramatically decreased in the shRNA group compared to the control group (decrease of approximately 70%) [41]. Consistently, the results of the present study showed that compared to sh-NUCB2 NC, sh-NUCB2#1 and sh-NUCB2#2 significantly decreased the mRNA expression of *NUCB2* and the protein expression of nesfatin-1, indicating a good silencing effect. The results of CCK-8 and EdU assays indicated that inhibition of NUCB2 markedly repressed RASF proliferation. The results of the wound healing and transwell assays demonstrated that inhibition of NUCB2 significantly suppressed the migratory and invasive abilities of RASFs. These findings further illustrate the pathogenic role of NUCB2 in RASFs and suggest that it may serve as a potential target for RA therapy.

This study has some limitations. First, although NUCB2 was mainly expressed in the lining layers of the RA synovium, where synovial fibroblasts are located, further studies are needed to assess the expression of NUCB2 in various types of synovial tissue cells. Second, a lack of in vivo experiments to confirm the functional role of NUCB2 in the tumorlike transformation of RASFs is a limitation of the present study. Third, the effect of NUCB2 on SFs should be explored in other control cells (osteoarthritis-SFs) to clarify the specificity of the effect of NUCB2 in RASFs.

Herein, we would also like to suggest potential future research directions. The present study was a preliminary study focusing on the effect of NUCB2 on the tumorlike transformation of RASFs in vitro. Further study should be conducted to confirm the role of NUCB2 in RASFs in collagen-induced arthritis (CIA) mice, an RA mouse model. Moreover, a Gene Ontology study using RNA sequencing and mass spectrometry for transcriptomic and proteomic analyses of RASFs after NUCB2 knockdown should be performed. Furthermore, mechanistic studies have shown that NUCB2/nesfatin-1 is able to stimulate Ca^2+^ inflow through L-type, N-type, or P/Q-type Ca^2+^ channels [42–44]. Considering that altered Ca^2+^ signaling is associated with critical events during tumor progression, such as proliferation, migration, invasion, and metastasis [45], it would be interesting to investigate whether Ca^2+^ signaling is involved in the specific mechanism by which NUCB2 promotes RASF proliferation, migration, and invasion.

## Conclusion

In conclusion, we found that NUCB2 knockdown suppressed RASF proliferation, migration, and invasion in vitro. Although our study highlights the role of NUCB2 in the pathogenesis of RA, additional in vivo and in vitro studies are recommended, as the NUCB2/nesfatin-1 receptor has not yet been identified.

## Data Availability

All data generated or analyzed during this study are included in this published article and its supplementary information files.

## References

[1] Firestein G (2003). Evolving concepts of rheumatoid arthritis. Nature.

[2] Müller-Ladner U, Ospelt C, Gay S, Distler O, Pap T (2007). Cells of the synovium in rheumatoid arthritis. Synovial fibroblasts. Arthritis Res Ther.

[3] Perlman H, Pope R (2010). The synovial lining micromass system: toward rheumatoid arthritis in a dish?. Arthritis Rheum.

[4] Tsuchiya H, Ota M, Fujio K (2021). Multiomics landscape of synovial fibroblasts in rheumatoid arthritis. Inflamm Regen.

[5] Bartok B, Firestein G (2010). Fibroblast-like synoviocytes: key effector cells in rheumatoid arthritis. Immunol Rev.

[6] Huber L, Distler O, Tarner I, Gay R, Gay S, Pap T (2006). Synovial fibroblasts: key players in rheumatoid arthritis. Rheumatology (Oxford).

[7] Oh-I S, Shimizu H, Satoh T, Okada S, Adachi S, Inoue K, Eguchi H, Yamamoto M, Imaki T, Hashimoto K, Tsuchiya T, Monden T, Horiguchi K, Yamada M, Mori M (2006). Identification of nesfatin-1 as a satiety molecule in the hypothalamus. Nature.

[8] Weibert E, Hofmann T, Stengel A (2019). Role of nesfatin-1 in anxiety, depression and the response to stress. Psychoneuroendocrinology.

[9] Dore R, Levata L, Lehnert H, Schulz C (2017). Nesfatin-1: functions and physiology of a novel regulatory peptide. J Endocrinol.

[10] Kmiecik A, Dzięgiel P, Podhorska-Okołów M (2021). Nucleobindin-2/nesfatin-1—a new cancer related molecule?. Int J Mol Sci.

[11] Skorupska A, Lenda R, Ożyhar A, Bystranowska D (2021). The multifaceted nature of nucleobindin-2 in carcinogenesis. Int J Mol Sci.

[12] Kan J, Yen M, Wang J, Wu D, Chiu Y, Ho Y (2016). Nesfatin-1/nucleobindin-2 enhances cell migration, invasion, and epithelial-mesenchymal transition via LKB1/AMPK/TORC1/ZEB1 pathways in colon cancer. Oncotarget.

[13] Tao R, Niu W, Dou P, Ni S, Yu Y, Cai L (2020). Nucleobindin-2 enhances the epithelial-mesenchymal transition in renal cell carcinoma. Oncol Lett.

[14] Takagi K, Miki Y, Tanaka S, Hashimoto C, Watanabe M, Sasano H (2016). Nucleobindin 2 (NUCB2) in human endometrial carcinoma: a potent prognostic factor associated with cell proliferation and migration. Endocr J.

[15] Zhang S, Rong G, Xu Y, Jing J (2021). Elevated nesfatin-1 level in synovium and synovial fluid is associated with pro-inflammatory cytokines in patients with rheumatoid arthritis. Int J Gen Med.

[16] Broeren M, de Vries M, Bennink M, Arntz O, Blom A, Koenders M (2016). Disease-regulated gene therapy with anti-inflammatory interleukin-10 under the control of the CXCL10 promoter for the treatment of rheumatoid arthritis. Hum Gene Ther.

[17] Woetzel D, Huber R, Kupfer P, Pohlers D, Pfaff M, Driesch D (2014). Identification of rheumatoid arthritis and osteoarthritis patients by transcriptome-based rule set generation. Arthritis Res Ther.

[18] Ungethuem U, Haeupl T, Witt H, Koczan D, Krenn V, Huber H (2010). Molecular signatures and new candidates to target the pathogenesis of rheumatoid arthritis. Physiol Genomics.

[19] Subramanian A, Tamayo P, Mootha V, Mukherjee S, Ebert B, Gillette M, Paulovich A, Pomeroy S, Golub T, Lander E, Mesirov J (2005). Gene set enrichment analysis: a knowledge-based approach for interpreting genome-wide expression profiles. Proc Natl Acad Sci USA.

[20] Tao J, Lu Z, Su J, Qian X, Zhang Y, Xu Y, Song S, Hang X, Peng X, Chen F (2021). ASIC1a promotes the proliferation of synovial fibroblasts via the ERK/MAPK pathway. Lab Investig.

[21] Zhang Y, Qian X, Yang X, Niu R, Song S, Zhu F, Zhu C, Peng X, Chen F (2020). ASIC1a induces synovial inflammation via the Ca/NFATc3/RANTES pathway. Theranostics.

[22] Vasileiadis G, Lundell A, Zhang Y, Andersson K, Gjertsson I, Rudin A (2021). Adipocytokines in untreated newly diagnosed rheumatoid arthritis: association with circulating chemokines and markers of inflammation. Biomolecules..

[23] Neumann E, Hasseli R, Ohl S, Lange U, Frommer K, Müller-Ladner U (2021). Adipokines and autoimmunity in inflammatory arthritis. Cells..

[24] Otero M, Lago R, Gomez R, Lago F, Dieguez C, Gómez-Reino J (2006). Changes in plasma levels of fat-derived hormones adiponectin, leptin, resistin and visfatin in patients with rheumatoid arthritis. Ann Rheum Dis.

[25] Seven A, Güzel S, Aslan M, Hamuryudan V (2009). Serum and synovial fluid leptin levels and markers of inflammation in rheumatoid arthritis. Rheumatol Int.

[26] Migita K, Maeda Y, Miyashita T, Kimura H, Nakamura M, Ishibashi H (2006). The serum levels of resistin in rheumatoid arthritis patients. Clin Exp Rheumatol.

[27] Xu Y, Chen F (2020). Antioxidant, anti-inflammatory and anti-apoptotic activities of nesfatin-1: a review. J Inflamm Res.

[28] Kvlividze T, Zavodovsky B, Akhverdyan Y, Polyakova Y, Sivordova L, Yakovlev A, Zborovskaya I (2019). Serum nesfatin-1 as a marker of systemic inflammation in rheumatoid arthritis. Klinicheskaia laboratornaia diagnostika.

[29] Robinson C, Tsang L, Solomon A, Woodiwiss A, Gunter S, Mer M (2018). Nesfatin-1 and visfatin expression is associated with reduced atherosclerotic disease risk in patients with rheumatoid arthritis. Peptides.

[30] Zhang H, Qi C, Li L, Luo F, Xu Y (2013). Clinical significance of NUCB2 mRNA expression in prostate cancer. J Exp Clin Cancer Res: CR.

[31] Kan J, Yen M, Wang J, Wu D, Chiu Y, Ho Y, Kuo P (2016). Nesfatin-1/nucleobindin-2 enhances cell migration, invasion, and epithelial-mesenchymal transition via LKB1/AMPK/TORC1/ZEB1 pathways in colon cancer. Oncotarget.

[32] Suzuki S, Takagi K, Miki Y, Onodera Y, Akahira J, Ebata A, Ishida T, Watanabe M, Sasano H, Suzuki T (2012). Nucleobindin 2 in human breast carcinoma as a potent prognostic factor. Cancer Sci.

[33] Liu G, Xu Z, Ma H (2018). Nesfatin-1/nucleobindin-2 is a potent prognostic marker and enhances cell proliferation. Migr Invasion Bladder Cancer Dis Markers.

[34] Zhang D, Lin J, Chao Y, Zhang L, Jin L, Li N (2018). Regulation of the adaptation to ER stress by KLF4 facilitates melanoma cell metastasis via upregulating NUCB2 expression. J Exp Clin Cancer Res: CR.

[35] Burgos J, Iresjö B, Smedh U (2016). MCG101-induced cancer anorexia-cachexia features altered expression of hypothalamic Nucb2 and Cartpt and increased plasma levels of cocaine- and amphetamine-regulated transcript peptides. Oncol Rep.

[36] Scott D, Wolfe F, Huizinga T (2010). Rheumatoid arthritis. Lancet (London, England).

[37] Zhu J, Thompson C (2019). Metabolic regulation of cell growth and proliferation. Nat Rev Mol Cell Biol.

[38] Najafi M, Farhood B, Mortezaee K (2019). Extracellular matrix (ECM) stiffness and degradation as cancer drivers. J Cell Biochem.

[39] Kai F, Drain A, Weaver V (2019). The extracellular matrix modulates the metastatic journey. Dev Cell.

[40] Romer L, Birukov K, Garcia J (2006). Focal adhesions: paradigm for a signaling nexus. Circ Res.

[41] Zhao J, Yun X, Ruan X, Chi J, Yu Y, Li Y, Zheng X, Gao M (2019). High expression of NUCB2 promotes papillary thyroid cancer cells proliferation and invasion. Onco Targets Ther.

[42] Brailoiu G, Dun S, Brailoiu E, Inan S, Yang J, Chang J, Dun N (2007). Nesfatin-1: distribution and interaction with a G protein-coupled receptor in the rat brain. Endocrinology.

[43] Iwasaki Y, Nakabayashi H, Kakei M, Shimizu H, Mori M, Yada T (2009). Nesfatin-1 evokes Ca^2+^ signaling in isolated vagal afferent neurons via Ca^2+^ influx through N-type channels. Biochem Biophys Res Commun.

[44] Ishida E, Hashimoto K, Shimizu H, Okada S, Satoh T, Kato I, Yamada M, Mori M (2012). Nesfatin-1 induces the phosphorylation levels of cAMP response element-binding protein for intracellular signaling in a neural cell line. PLoS ONE.

[45] Monteith G, McAndrew D, Faddy H, Roberts-Thomson S (2007). Calcium and cancer: targeting Ca2+ transport. Nat Rev Cancer.

